# MHIF-MSEA: a novel model of miRNA set enrichment analysis based on multi-source heterogeneous information fusion

**DOI:** 10.3389/fgene.2024.1375148

**Published:** 2024-03-22

**Authors:** Jianwei Li, Xuxu Ma, Hongxin Lin, Shisheng Zhao, Bing Li, Yan Huang

**Affiliations:** ^1^ Institute of Computational Medicine, School of Artificial Intelligence, Hebei University of Technology, Tianjin, China; ^2^ Key Laboratory of Carcinogenesis and Translational Research (Ministry of Education, Beijing), Department of Anesthesiology, Peking University Cancer Hospital and Institute, Beijing, China

**Keywords:** miRNA, functional enrichment analysis, miRNA-miRNA association network, random walk with restart, heterogeneous information fusion

## Abstract

**Introduction:** MicroRNAs (miRNAs) are a class of non-coding RNA molecules that play a crucial role in the regulation of diverse biological processes across various organisms. Despite not encoding proteins, miRNAs have been found to have significant implications in the onset and progression of complex human diseases.

**Methods:** Conventional methods for miRNA functional enrichment analysis have certain limitations, and we proposed a novel method called MiRNA Set Enrichment Analysis based on Multi-source Heterogeneous Information Fusion (MHIF-MSEA). Three miRNA similarity networks (miRSN-DA, miRSN-GOA, and miRSN-PPI) were constructed in MHIF-MSEA. These networks were built based on miRNA-disease association, gene ontology (GO) annotation of target genes, and protein-protein interaction of target genes, respectively. These miRNA similarity networks were fused into a single similarity network with the averaging method. This fused network served as the input for the random walk with restart algorithm, which expanded the original miRNA list. Finally, MHIF-MSEA performed enrichment analysis on the expanded list.

**Results and Discussion:** To determine the optimal network fusion approach, three case studies were introduced: colon cancer, breast cancer, and hepatocellular carcinoma. The experimental results revealed that the miRNA-miRNA association network constructed using miRSN-DA and miRSN-GOA exhibited superior performance as the input network. Furthermore, the MHIF-MSEA model performed enrichment analysis on differentially expressed miRNAs in breast cancer and hepatocellular carcinoma. The achieved *p*-values were 2.17e^(-75)^ and 1.50e^(-77)^, and the hit rates improved by 39.01% and 44.68% compared to traditional enrichment analysis methods, respectively. These results confirm that the MHIF-MSEA method enhances the identification of enriched miRNA sets by leveraging multiple sources of heterogeneous information, leading to improved insights into the functional implications of miRNAs in complex diseases.

## 1 Introduction

MicroRNAs (miRNAs) are a type of non-coding RNAs (ncRNAs) that are approximately 22 nucleotides long. There is growing to suggest that miRNAs play a crucial regulatory role in human biological processes (Slack and Chinnaiyan, 2019). Non-coding RNAs are involved in the regulation of gene expression in various physiological activities, including cell proliferation (Cheng et al., 2005), cell differentiation (Miska, 2005), apoptosis (Xu et al., 2004), immune function modulation in animals (Stern-Ginossar et al., 2007), and gene expression level regulation (Shivdasani, 2006). Furthermore, they are closely associated with the occurrence and development of complex human diseases (Esteller, 2011). Due to the rapid development and widespread application of high-throughput sequencing technologies, researchers have accumulated a wealth of information regarding miRNA attributes. High-throughput experimental analysis have indicated that miRNAs often exert their regulatory functions as sets rather than individual molecules (Yoshida et al., 2021). Consequently, there has been a shift from analyzing the functional roles of individual miRNAs to a comprehensive analyzing the collective functions of miRNA sets, highlighting the importance of functional enrichment analysis methods. However, accurately analyzing the relationships among miRNAs within a set and understanding the underlying biological mechanisms remain challenging (Wang and Krishnan, 2014; Fillinger et al., 2019). To data, several miRNAs set functional enrichment analysis methods have been developed, which can be divided into two categories based on their data sources and execution algorithms.

The first category for inferring the functionality of miRNA sets is based on miRNA target genes. These methods generally involve three main steps. First, a set of differentially expressed miRNAs is chosen based on experimental results or select miRNAs of interest to form an input miRNA list. Second, high-confidence miRNA target databases or miRNA target prediction tools are used to identify miRNA target genes. Third, gene enrichment analysis methods are employed to uncover enriched pathways, functions, or phenotypes of the target genes, thereby inferring the potential functions of the miRNA set. One example of this category is miRPath (Papadopoulos et al., 2009), which is one of the earliest methods released. It has been updated to miRPath V3.0 which utilizes the TarBase database, microT-CDS, and TargetScan algorithm to obtain a collection of miRNA target genes. It performs functional enrichment analysis of miRNA target genes based on the Gene Ontology (GO) database and the Kyoto Encyclopedia of Genes and Genomes (KEGG) signaling pathway database. MiRPath V3.0 not only employs traditional hypergeometric distribution to calculate enrichment results but also introduces an unbiased empirical distribution and an improved meta-analysis statistical method. The tool also supports reverse searching, allowing for the discovery that regulate miRNAs regulating genes within specified GO terms or pathways. Additionally, it can perform functional enrichment analysis for miRNAs from seven different species. Another tool in this category is MiRWalk (Sticht et al., 2018), which integrates miRNA target gene prediction and retrieval of miRNA and gene interaction information. It can also perform functional enrichment analysis of miRNA target genes. Its latest data was updated in January 2022. MiRWalk acquires experimentally validated miRNA target genes from the miRTarBase (Huang et al., 2022) and predicts miRNA target genes according to the TargetScan and miRDB. Finally, the GSEA method is used to conduct gene functional enrichment analysis and supports enrichment analysis based on the GO and KEGG databases. However, these methods have some notable limitations. A single miRNA may be associated with hundreds of genes, which can lead to the inclusion of multiple pathways that are only weakly correlated with the user-input miRNA. This would result in a substantial bias in the enrichment analysis results (Bleazard et al., 2015; Godard and van Eyll, 2015).

The second category of methods involves performing functional enrichment analysis directly on miRNAs based on miRNA sets in a background knowledge database. These miRNA background sets represent a group of miRNAs that share certain common biological characteristics. With the continuous advancement of miRNA research, numerous resources of miRNA background sets have emerged. Consequently, several methods and tools have been developed to directly perform enrichment analysis on miRNAs, leading to significant achievements in this field. TAM (Lu et al., 2010) is the first tool to conduct functional enrichment analysis directly on miRNAs using a knowledge base of miRNA sets. TAM included 362 miRNA sets, comprising 43 miRNA functional sets and 183 miRNA disease sets. It utilized the hypergeometric distribution test to assess whether the user-input miRNA list was enriched in each miRNA set. In 2018, we updated the TAM and proposed TAM 2.0 (Li et al., 2018). By employing manual collection and annotation, TAM 2.0 expanded the miRNA sets based on an extensive review of the relevant literature. It now includes 547 miRNA disease sets and 158 miRNA functional sets. Currently, TAM 2.0 stands as the most comprehensive knowledge base of miRNA sets, providing researchers with a valuable resource for performing functional enrichment analysis on miRNA lists of interest. Another miRNA functional enrichment analysis tool is MiEAA (Backes et al., 2016), which supports multiple species and is set to release its second edition in 2020. MiEAA conducts functional enrichment analysis not only on miRNA precursors but also on mature miRNAs. The knowledge base for miRNA precursors includes miRNA clusters, miRNA families, miRNA chromosome locations and conserved miRNA sets. Similarly, the knowledge base for mature miRNAs is derived from miRNA target gene functions and pathways.

In this study, we introduced a novel model, the miRNA Functional Enrichment Analysis model based on Multisource Heterogeneous Information Fusion (MHIF-MSEA), with the aim of delving into the regulatory function of miRNAs in greater detail. MHIF-MSEA employs a multi-step approach to construct an integrated network that combines diverse sources of information. Initially, a disease-associated miRNA similarity algorithm was employed to establish a disease-associated miRNA similarity network. Subsequently, the miRNA similarity algorithm (MIRGOFS) utilized the miRNA similarity algorithm (MIRGOFS) to generate a miRNA similarity network based on the Gene Ontology (GO) annotations of target genes. Additionally, a miRNA similarity network by considering the target genes and their association with miRNAs was constructed, taking into account the protein-protein interactions resulting from the transcriptional activity of these target genes. By performing pairwise and comprehensive fusion of the aforementioned three networks, we derived a fused miRNA-miRNA association network that integrates heterogeneous information sources. This fused network served as a basis for exploring miRNA nodes closely associated with the miRNAs in the user-input miRNA list. To achieve this, a random walk with restart algorithm was employed, effectively expanding the user-input miRNAs according to the fused miRNA-miRNA association network.

To showcase the effectiveness and accuracy of the MHIF-MSEA model, three case were introduced, which were focused on colon cancer, breast cancer, and hepatocellular carcinoma. The experimental results demonstrated that the miRNA-miRNA association network constructed with miRSN-DA and miRSN-GOA constitutes the optimal input network. Subsequently, the MHIF-MSEA model conducts enrichment analysis on differentially expressed miRNAs in breast cancer and hepatocellular carcinoma. The obtained *p*-values for these analyses were $2.17e^{-75}$ and $1.50e^{-77}$ , respectively. Moreover, the hit rates were improved by 39.01% and 44.68%, respectively. These results substantiate the usefulness of the MHIF-MSEA model in further exploring the functional characteristics of the user-input miRNA list. The source code and experimental data for MHIF-MSEA are available at https://github.com/awesomero/MHIF-MSEA.

## 2 Materials and methods

### 2.1 Datasets

In this study, experimentally validated human miRNA-disease association data were obtained from the HMDD v4.0 database (Cui et al., 2023), which included 18,732 unique miRNA-disease associations involving 1,206 miRNAs and 892 diseases. Subsequently, the similarity between 1,041 miRNAs was derived from the MISIM v2.0 (Li et al., 2019) web server (see Supplementary Table S1). In addition, the similarity between 1,063 miRNAs was calculated using the MIRGOFS method (see Supplementary Table S2). A protein-protein interaction network (PPIN) containing 11,305 proteins and 69,331 protein-protein interactions was obtained from the MINT database (Chatr-aryamontri et al., 2006). In addition, miRNA target gene data validated by rigorous experimental methods such as reporter assay and western blot were downloaded from the miRTarbase v9.0 database (Huang et al., 2022). The mature miRNA format was uniformly converted to the precursor miRNA format, and a dataset of 10,130 miRNA-target gene pairs involving 677 miRNA precursors was obtained. Based on the above data, the similarity between 495 miRNAs was obtained in our study (see Supplementary Table S3).

### 2.2 MiRNA similarity network based on miRNA-disease associations

For each disease name represented by MeSH, it can be represented as a Directed Acyclic Graph (DAG). For a given disease, denoted as d , it can be expressed in the DAG using Eq. 1:

$$DAG_{d}=\left(d,T_{d},E_{d}\right)$$ (1)

where ${\;T}_{d\;}$ is the set of nodes composed of d and all its ancestor nodes, and ${\;E}_{d}$ denotes the set of all edges in the graph. In the DAG graph of disease d , the semantic contribution value D of disease t to disease d can be defined using Eq. 2.

$$\left\{\begin{array}{l} D_{d}\left(d\right)=1\\ D_{d}\left(t\right)=\max\left\{∆*D_{d}\left(t^{\prime }\right)|t^{\prime }\in \text{children of }t\right\}\text{ if }t\neq d \end{array}\right.$$ (2)

Equation 2 indicates that selecting the shortest path is a necessary step for obtaining the maximum semantic contribution value when there are multiple paths from disease t to disease d in the DAG graph. Here, ∆ is the semantic contribution decay factor, which reflects the influence of parent nodes on child nodes in the DAG structure, with an initial value between 0 and 1. According to the related research (Xuan et al., 2013), ∆ is usually set to 0.5. By summing up the semantic contribution values of all nodes in the DAG, the semantic contribution value of a given disease d can be obtained, and it is shown in Eq. 3.

$$DV\left(d\right)=\sum_{t\in T_{d}}D_{d}\left(t\right)$$ (3)

With the semantic contribution values for each disease, the similarity $S\left(d_{i},d_{j}\right)$ between any two diseases ${\;d}_{i}$ and $d_{j}$ can be calculated according to Eq. 4:

$$S\left(d_{i},d_{j}\right)=\frac{\sum_{t\in T_{d_{i}}\cap T_{d_{j}}}\;\left(D_{d_{i}}\left(t\right)+D_{d_{j}}\left(t\right)\right)}{DV\left(d_{i}\right)+DV\left(d_{j}\right)}$$ (4)

where t is the diseases associated with disease ${\;d}_{i}$ and $d_{j}$ simultaneously in the DAG structure, $\text{DV}\left(d_{i}\right)$ and $\text{DV}\left(d_{j}\right)$ represent the semantic contribution values of diseases ${\;d}_{i}$ and $d_{j}$ respectively, $D_{d_{i}}\left(t\right)$ and $D_{d_{j}}\left(t\right)$ denote the semantic contribution values of disease t to diseases ${\;d}_{i}$ and $d_{j}$ respectively.

Next, the functional similarity between miRNAs based on disease associations was calculated using the MISIM (Wang et al., 2010). The core idea of the MISIM algorithm is that the similarity between miRNAs can be obtained by calculating the similarity between the diseases associated with the two miRNAs. The MISIM algorithm defines a set of diseases as $D=\left(d_{1},d_{2},\cdots d_{n}\right)$ . The similarity $S\left(d,D\right)$ between a disease d and D was the maximum of the semantic similarity between disease d and one disease in the set D , as shown in Eq. 5:

$$S\left(d,D\right)=\max_{1\leq i\leq m}\left(S\left(d,d_{i}\right)\right)$$ (5)

where m is the number of diseases in the disease set D , $d_{i}$ is a disease in the disease set, $d_{i}\in D$ , and $Sim\left(d,d_{i}\right)$ is the similarity between diseases d and ${\;d}_{i}$ . For two miRNAs, $m_{1\;}$ and ${\;m}_{2}$ , their functional similarity can be expressed by Eq. 6:

$$MISIM\left(m_{1},m_{2}\right)=\frac{\sum_{1\leq i\leq m}S\left(d_{1i},D_{2}\right)+\sum_{1\leq j\leq n}S\left(d_{2j},D_{1}\right)}{m+n}$$ (6)

where $D_{1\;}$ and $D_{2}$ represent the sets of diseases associated with ${\;m}_{1}$ and ${\;m}_{2}$ , m and n represent the number of diseases in sets $D_{1}$ and $D_{2}$ , ${\;d}_{1i}$ and ${\;d}_{2j}$ represent specific disease elements in the sets $D_{1}$ and $D_{2}$ , respectively.

The latest iteration, MISIM v2.0, represents an enhanced version that incorporates an expanded collection of miRNA-disease associations. This updated version not only enables more accurate predictions but also encompasses a broader spectrum of miRNA similarities. In our study, we leveraged this resource to construct a disease-based miRNA similarity network. Additionally, the semantic similarity between diseases was utilized as well as the MISIM v2.0 method to calculate the similarity between pairs of miRNAs. Specifically, we obtained the similarity scores for 1,041 miRNAs through this approach.

### 2.3 MiRNA similarity network based on GO annotations of target genes

MIRGOFS was designed to calculate the functional similarity of miRNAs by utilizing the Gene Ontology (GO) annotations of their target genes (Yang et al., 2018). Unlike previous approaches, MIRGOFS considered the entire set of GO annotations for target genes as a whole, associating each miRNA with a GO set that may contain redundant terms. The similarity between two miRNAs was then determined by assessing the similarity between their respective GO sets. Notably, MIRGOFS demonstrated superior prediction accuracy compared to existing methods.

MIRGOFS first calculates the semantic similarity between Gene Ontology (GO) terms, which can be represented graphically by directed acyclic graphs (DAGs). The semantic similarity between GO terms, as shown in Eq. 7.

$$Sim\left(x,y\right)=\left(\frac{IC\left(L_{x,y}\right)}{IC\left(x\right)}+\frac{IC\left(L_{x,y}\right)}{IC\left(y\right)}+\frac{IC\left(L_{x,y}\right)}{IC\left(H_{x,y}\right)}\right)\times \left(IC\left(x\right)+IC\left(y\right)\right)$$ (7)

The specific calculation for the Information Content (IC) was shown in Eq. 8.

$$IC\left(C\right)=-\log\left(\frac{\left|G_{x}\right|}{\left|G_{root\;}\right|}\right)$$ (8)

where x is a GO term, $G_{x\;}$ is the set of genes associated with term x and $G_{\text{root}\;}$ is the set of genes related to the root node. $L_{x,y}$ and ${\;H}_{x,y}$ denote the sets of Lowest Common Ancestors (LCA) and Highest Common Descendants (HCD) for the pair $\left(x,y\right)$ , respectively.

For the calculation of miRNA similarity, MiRGOFS has also undergone relevant improvements. The weight ${\;w}_{x}$ of each GO term is calculated based on the cumulative hypergeometric distribution. Assuming that there are N genes annotated with GO terms in the database, with M genes annotated with term x and for a given miRNA with n target genes, where k genes are annotated with term x , the following Eq. 9 holds.

$$w_{x}=-\log\left(1-\sum_{i=0}^{k-1}\;\frac{C_{M}^{i}\times C_{N-M}^{n-i}}{C_{N}^{n}}\right)$$ (9)

Finally, the similarity between the sums of two miRNAs was calculated based on the weighted Euclidean distance, as shown in Eq. 10:

$$Sim_{m_{q},m_{t}}=\sqrt{\sum_{i=1}^{n}\;{\left(Sim\left(x,B\right)\times w_{x}\right)}^{2}}$$ (10)

where n is the number of non-redundant GO terms associated with miRNA ${\;m}_{q}$ and B is the set of GO terms associated with miRNA ${\;m}_{t}$ . In this study, the MIRGOFS method was employed to calculate the similarity between 1,063 miRNAs.

### 2.4 MiRNA similarity network based on protein-protein interactions of target genes

Protein is the product of miRNA target genes. The miRFunSim method was proposed based on graph theory (Sun et al., 2013), utilizing the protein-protein interaction network (PPIN) and the association between miRNA and target genes to predict the functional similarity between miRNAs. Given two miRNAs of interest, denoted as $m_{1}$ and $m_{2}$ , the miRFunSim method first obtained the target gene lists for each miRNA. These target genes were then mapped onto the PPIN, creating a subnetwork of PPIN relationships of the target genes. Finally, the shortest path lengths between the target genes were calculated as the distances between them. The functional similarity between $m_{1}$ and $m_{2}$ was defined as the inverse of the average pairwise distance between their target gene lists. The specific calculation equation was expressed in Eq. 11:

$$miRFunSim\left(m_{1},m_{2}\right)=\frac{N}{\sum_{i\in T_{m_{1}}}\;\sum_{j\in T_{m_{2}}}\;Dist\left(i,j\right)}$$ (11)

where ${\;T}_{m_{1}}$ and $T_{m_{2}}$ represent the target gene lists for $m_{1}$ and $m_{2}$ , respectively. The variables i and j denote specific genes within the sets ${\;T}_{m_{1}}$ and ${\;T}_{m_{2}}$ , n is the number of paths in the PPIN subnetwork, and $Dist\left(i,j\right)$ is the distance between target genes i and j . Applying the aforementioned calculation methods to the two datasets mentioned earlier, a miRNA similarity network based on protein-protein interactions with target genes was obtained, comprising 495 miRNAs.

### 2.5 Construction of a miRNA-miRNA association network

In this section, three miRNA similarity networks (miRSN-DA, miRSN-GOA, and miRSN-PPI) were fused using the averaging method, and the resulting fused similarity network served as the miRNA-miRNA association network. For example, the fusion process of miRSN-DA and miRSN-GOA, is shown as follows. Firstly, the intersection of the miRNAs from these two miRNA similarity matrices was obtained. It enabled us to identify the common miRNAs present in both networks. Secondly, the two miRNA similarity matrices individually by removing the similarity information for miRNAs were processed that did not appear in the intersection. Consequently, two miRNA similarity matrices with matching row and column dimensions were obtained. Thirdly, the two similarly shaped miRNA similarity matrices were averaged to generate a fused similarity matrix exclusively for the intersecting miRNAs. Finally, we incorporated the similarity information for miRNAs that existed only in the individual matrices into the fused similarity matrix. This integration step allowed us to achieve the desired fused miRNA similarity matrix for the miRSN-DA and miRSN-GOA networks. By following this fusion procedure, the miRSN-DA and miRSN-GOA networks were successfully merged, and analogous steps were applied to fuse other miRNA similarity networks as well.

### 2.6 Random walk with restart

The random walk with restart algorithm is an effective network analysis algorithm that captures node proximity within a network. It has found widespread application in bioinformatics (Valdeolivas et al., 2019). In this study, we employed this method to identify closely related nodes in the miRNA-miRNA association network. The algorithm initiates from a seed node in the network, and at each step, it randomly selects a neighboring node or returns to the original node. The ultimate objective of our study was to solve Eq. 12:

$$r=cWr+\left(1-c\right)e$$ (12)

where c is the restart probability, with a magnitude between $\left(0,1\right)$ . W is the transition probability matrix, each element $W_{ij}$ in the transition probability matrix represents the probability from node i to node j , in the miRNA-miRNA association network, it represents the similarity between two nodes, e is the initial vector, and r is the final vector. As the number of iteration increase, the r node will eventually converge, resulting in a probability score. A lower score indicates a weaker association with the seed node of the random walk. In this study, the differentially expressed miRNAs were employed as seed nodes for conducting the random walk with restart method on the miRNA-miRNA association network. The algorithm was executed until convergence was achieved. Subsequently, we identified the nodes with non-zero random walk probabilities, indicating their significance in the network. These nodes were considered as expanded miRNAs, and their collective information was utilized to generate the expanded miRNA list. To further enhance our analysis, the expanded miRNA list was merged with the user-input miRNA list. This merged list was then subjected to enrichment analysis using the MHIF-MSEA method. This comprehensive approach allowed us to explore the functional enrichment of the combined miRNA set and gain insights into their potential biological implications.

### 2.7 MHIF-MSEA model

Finally, a functional enrichment analysis model was developed based on the miRNA-miRNA association network, which integrated heterogeneous information from multiple sources. This model aimed to identify functional enrichment patterns associated with miRNAs. Instead of directly conducting functional enrichment analysis on the miRNA list, the model employed a mapping strategy. Initially, the nodes in the miRNA list were mapped onto the miRNA-miRNA association network, enabling the identification of seed nodes for subsequent random walks. The random walk with restart algorithm was then applied to explore network nodes closely connected to the seed nodes within the miRNA-miRNA association network. The algorithm iteratively converged until all nodes were considered. Following convergence, nodes with random walk probability scores in the top 50% were selected as a set closely associated with the miRNA list. These nodes were subsequently merged with the original miRNA list, generating an expanded miRNA list that encompassed both the seed nodes and the closely connected nodes from the miRNA-miRNA association network. To evaluate the functional enrichment of the expanded miRNA list, enrichment analysis was performed using the Over-Representation Analysis (ORA) method. This analysis technique enabled the identification of functional categories or biological processes that exhibited significant enrichment within the expanded miRNA list. By applying this comprehensive approach, valuable insights into the functional implications of the miRNA set were obtained. Regarding the selection of nodes obtained after the random walk with restart, we tried four ratios, 30%, 40%, 50%, and 60%. The experimental results show that the enrichment analysis effect of the 50% ratio is the best (see Supplementary Table S4).

In enrichment analysis, the incorporation of a curated knowledge base for miRNAs enhances the accuracy of the analysis. TAM 2.0, which has undergone extensive manual literature review, has substantially expanded the knowledge base, making it the most comprehensive and reliable server for conducting direct miRNA-based enrichment analysis. From TAM 2.0, we extracted a high-quality annotated miRNA collection knowledge base. This knowledge base consists of 1,412 miRNA collections, encompassing a total of 1,714 miRNA precursors. The meticulous curation process employed in TAM 2.0 ensures the reliability and thoroughness of the miRNA collection information. Utilizing this rich knowledge base enhances the quality and reliability of our enrichment analysis, providing a solid foundation for further investigations.

The miRNA functional enrichment analysis relies on the application of the hypergeometric distribution and *p*-value testing to assess the statistical significance of the overlap between the miRNA list of interest and the miRNA collection. By calculating the *p*-value, the level of statistical significance associated with the enrichment analysis was determined. A lower *p*-value indicates a more significant result, suggesting a higher enrichment of miRNAs within a specific pathway or category. In this analysis, the commonly adopted threshold for *p*-values is set at 0.05. This threshold helps determine whether the observed overlap between the miRNA list and the miRNA collection is statistically significant. If the calculated *p*-value is below this threshold, it suggests that the observed enrichment is unlikely to have occurred by chance alone. Thus, a *p*-value below 0.05 indicates a meaningful enrichment of miRNAs in the particular pathway or category under investigation. The formula for calculating the *p*-value is shown in Eq. 13:

$$P-\text{value}=\sum_{i=m}^{M}\;\frac{C_{M}^{i}C_{N-M}^{n-i}}{C_{N}^{n}}$$ (13)

where n is the number of miRNAs in the differentially expressed miRNA list or the miRNA list of interest, N is the total number of miRNAs in the reference miRNA collection, M is the number of miRNAs in a specific functional set, and m is the intersection set between the input miRNA list and the specific miRNA functional set. After deriving the P-value, the Benjamini-Hochberg method was used to calculate the corrected *p*-value, referred to as the False Discovery Rate (FDR), to mitigate the problem of false positives. Smaller *p*-values and FDR values indicate a more significant enrichment result, and an FDR threshold of < 0.05 is used as the criterion for filtering significantly enriched results.

**FIGURE 1:**
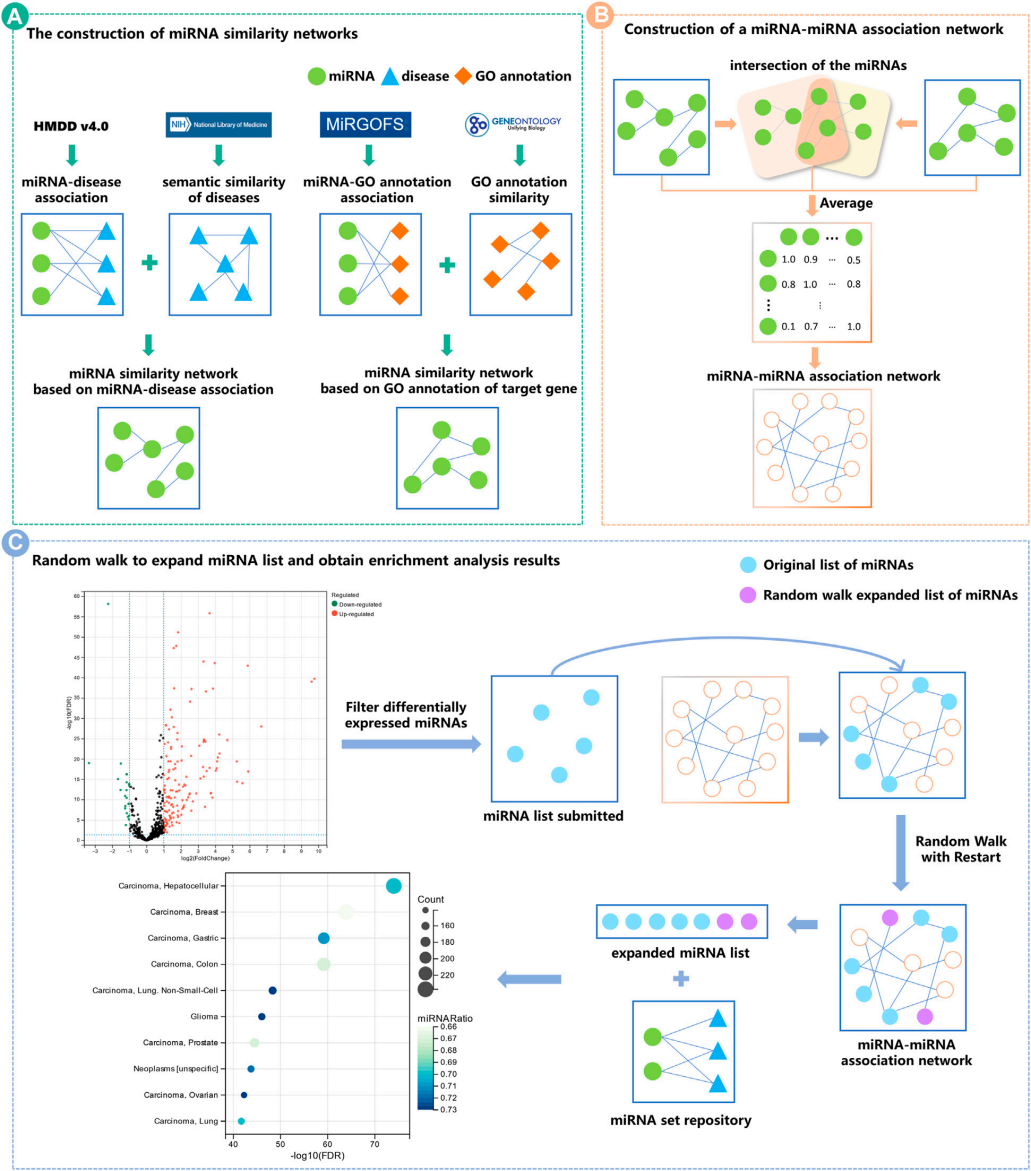
Flowchart of the MHIF-MSEA model. **(A)** Two miRNA similarity networks were constructed based on miRNA-disease associations and GO annotations of target genes, respectively. **(B)** Two miRNA similarity networks were fused by taking the average. The fused network served as the miRNA-miRNA association network for the MHIF-MSEA model. **(C)** The expanded miRNA list was obtained with network random walk with the restart method and merged with the original miRNA list, followed by functional enrichment analysis.

In this study, we developed a novel model called MHIF-MSEA, and the flowchart of it is shown in Figure 1.

## 3 Results

In this study, miRNA differential expression profiles for breast cancer, hepatocellular carcinoma, and colon cancer were collected and subjected to preprocessing steps. From these profiles, a list of differentially expressed miRNAs was extracted, specifically for subsequent case analysis investigations. In the comparative analysis and selection of fused miRNA similarity networks, we identified four distinct fusion networks of interest. These included the fusion of miRSN-DA and miRSN-GOA, the fusion of miRSN-DA and miRSN-PPI, the fusion of miRSN-GOA and miRSN-PPI, and the overall fusion of miRSN-DA, miRSN-GOA, and miRSN-PPI. To identify the optimal fused network for miRNA-miRNA association network, we contrasted the results of the enrichment analysis across the three different case scenarios. Following the selection of the optimal fused network, we conducted enrichment analysis on two specific cases: breast cancer and hepatocellular carcinoma. The MHIF-MSEA model was employed for this analysis. Subsequently, a detailed discussion of the experimental outcomes was presented, highlighting the implications of the results.

### 3.1 Collection of differential expression miRNA sets of diseases

**FIGURE 2:**
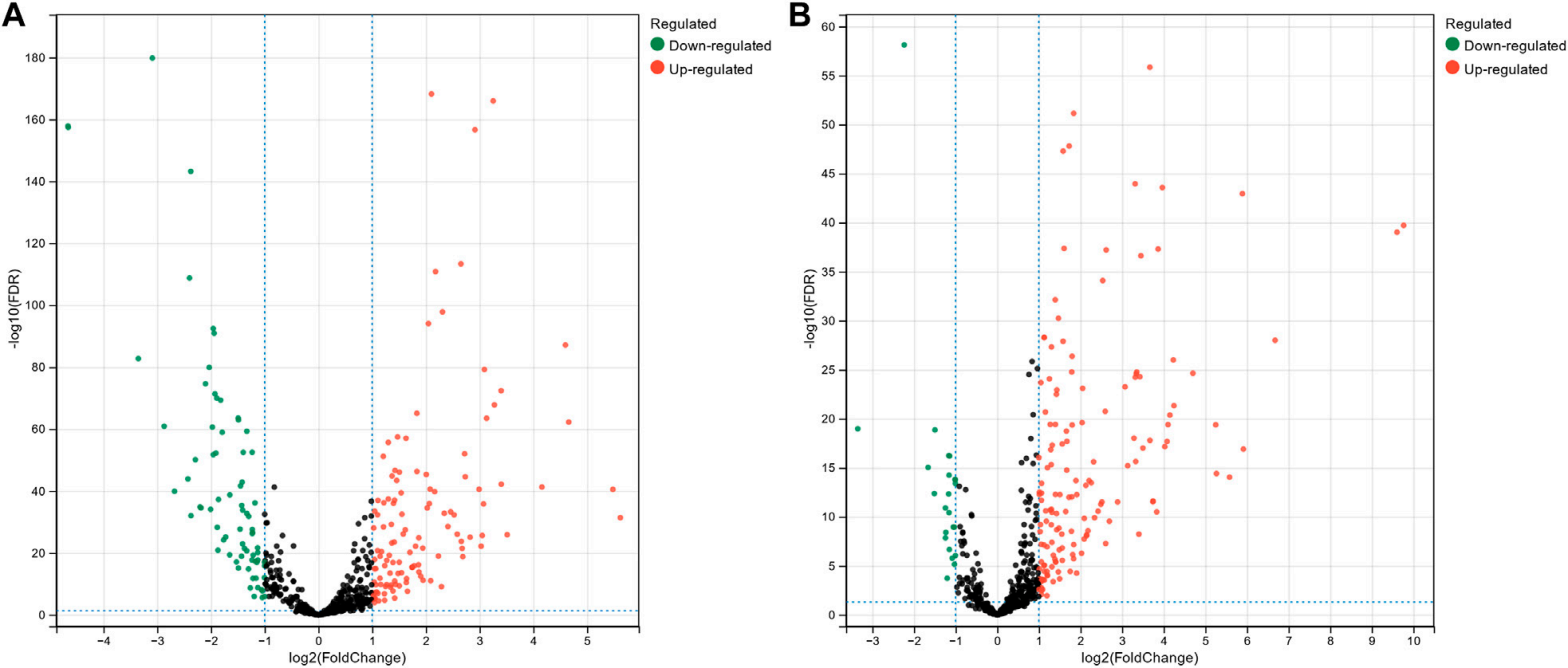
Volcano plot of differentially expressed miRNAs in breast cancer **(A)** and hepatocellular carcinoma **(B)**.

The miRNA expression profile data for breast cancer, hepatocellular carcinoma, and colon cancer were collected from The Cancer Genome Atlas Program (TCGA). Subsequently, we conducted differential expression analysis on these datasets to identify miRNAs that exhibited significant expression changes across different cancer types. This analysis aimed to uncover miRNAs that could potentially serve as biomarkers or play crucial roles in the development and progression of these cancers. The miRNA progenitor expression profile data for breast cancer were downloaded from TCGA, comprising 1,085 tumor tissue samples and 104 normal tissue samples. For hepatocellular carcinoma, the expression profile data consist of 374 tumor tissue samples and 50 normal tissue samples, while colon cancer expression profile data consisted of 195 tumor tissue samples and 198 normal tissue samples. Differential expression analysis of the downloaded miRNA expression profiles was performed using the DESeq2 package (Love et al., 2014) in the R platform. Figure 2 shows volcano plots illustrating differentially expressed miRNAs in breast cancer and hepatocellular carcinoma. The x-axis represents $\log_{2}FC$ , with larger absolute values indicating more significant differential expression of miRNAs. The y-axis represents ${-\log}_{10}\left(FDR\right)$ , with higher values indicating more significant differential expression of miRNAs. Green, red, and black dots in the figures represent miRNAs with low expression, high expression, and normal expression in tumor tissues, respectively. In breast cancer, 195 differentially expressed miRNAs were identified, including 121 upregulated and 74 downregulated miRNAs. For hepatocellular carcinoma, 175 differentially expressed miRNAs were identified, including 152 upregulated and 23 downregulated miRNAs. In colon cancer, 199 differentially expressed miRNAs were identified, including 142 upregulated and 57 downregulated miRNAs.

### 3.2 MiRNA-miRNA association network

In this study, four fusion scenarios were investigated, namely the fusion of miRSN-DA and miRSN-GOA, the fusion of miRSN-DA and miRSN-PPI, the fusion of miRSN-GOA and miRSN-PPI, and the total fusion of miRSN-DA, miRSN-GOA, and miRSN-PPI. The focus of our experiments was on three cancer cases: colon cancer, breast cancer, and hepatocellular carcinoma. The objective was to explore differentially expressed miRNAs within these cancer types. For each case, we employed single miRNA similarity networks and various fused miRNA similarity networks as input networks. These networks were then utilized in combination with random walk with restart algorithms to expand the original miRNA lists specific to each cancer type. We compared the performance of the enrichment analysis results when the similarity coefficient of the random walk with restart was 0.6, 0.7, 0.75, 0.8, and 0.85. In the miRNA-miRNA association network, the edges greater than the similarity coefficient were retained and those less than the similarity coefficient were deleted. The experimental results showed that choosing 0.6 as the similarity coefficient achieved the optimal effect for enrichment analysis (see Supplementary Table S5). Subsequently, enrichment analysis was conducted on the expanded miRNA lists. To compare the outcomes of the enrichment analysis, key evaluation metrics such as *p*-value, FDR value, and hit counts were employed. The results of the enrichment analysis for the three cases are presented in Tables 1–3. The first row of each table, labeled as “None,” represents the enrichment analysis performed on the original list of differentially expressed miRNAs. The subsequent rows illustrate the results of the enrichment analysis conducted on the expanded miRNA lists using the respective miRNA similarity networks. Within each table, the *p*-values, FDR values, and hit counts are specific to the enrichment analysis results corresponding to the particular cancer type mentioned. For instance, in Table 1, all the *p*-values, FDR values, and hit counts are associated with the “Carcinoma, Colon” entry in the enrichment analysis results. Similarly, in Table 2, these metrics pertain to the “Carcinoma, Breast” entry in the enrichment analysis results.

**TABLE 1:** Enrichment analysis results for the expanded differentially expressed miRNA lists in colon cancer using various miRNA similarity networks.

miRNA similarity network	*p*-value	FDR value	Hit counts
None	$3.22e^{-23}$	$1.01e^{-20}$	91
miRSN-DA	$6.41e^{-39}$	$1.37e^{-36}$	173
miRSN-GOA	$3.21e^{-31}$	$1.03e^{-28}$	170
miRSN-PPI	$1.82e^{-34}$	$5.83e^{-32}$	173
miRSN-DA + miRSN-GOA	$1.51e^{-55}$	$2.43e^{-53}$	204
miRSN-DA + miRSN-PPI	${4.28e}^{-37}$	$9.18e^{-35}$	171
miRSN-GOA + miRSN-PPI	$1.69e^{-30}$	$5.44e^{-28}$	170
miRSN-DA + miRSN-GOA	$1.67e^{-50}$	$2.69e^{-48}$	198
+ miRSN-PPI			

**TABLE 2:** Enrichment analysis results for the expanded differentially expressed miRNA lists in breast cancer using various miRNA similarity networks.

miRNA similarity network	*p*-value	FDR value	Hit counts
None	$2.92e^{-29}$	$6.11e^{-27}$	103
miRSN-DA	$2.44e^{-47}$	$7.84e^{-45}$	193
miRSN-GOA	$1.18e^{-32}$	$1.89e^{-30}$	182
miRSN-PPI	$2.28e^{-36}$	$3.66e^{-34}$	187
miRSN-DA + miRSN-GOA	$2.17e^{-75}$	$6.95e^{-73}$	238
miRSN-DA + miRSN-PPI	$6.81e^{-47}$	$2.18e^{-44}$	194
miRSN-GOA + miRSN-PPI	$1.68e^{-29}$	$2.07e^{-27}$	179
miRSN-DA + miRSN-GOA	$3.92e^{-75}$	$1.62e^{-72}$	238
+ miRSN-PPI			

**TABLE 3:** Enrichment analysis results for the expanded differentially expressed miRNA lists in hepatocellular carcinoma using various miRNA similarity networks.

miRNA similarity network	*p*-value	FDR value	Hit counts
None	$2.81e^{-22}$	$1.74e^{-19}$	86
miRSN-DA	$3.81e^{-52}$	$2.44e^{-49}$	196
miRSN-GOA	$1.37e^{-32}$	$4.39e^{-30}$	181
miRSN-PPI	$1.65e^{-30}$	$5.31e^{-28}$	174
miRSN-DA + miRSN-GOA	$1.50e^{-77}$	$9.61e^{-75}$	237
miRSN-DA + miRSN-PPI	${6.29e}^{-48}$	$4.05e^{-45}$	193
miRSN-GOA + miRSN-PPI	$5.61e^{-33}$	$3.60e^{-30}$	183
miRSN-DA + miRSN-GOA	$3.44e^{-75}$	$2.21e^{-72}$	234
+ miRSN-PPI			

The colon cancer miRNA set consisted of 315 miRNAs, out of which only 91 miRNAs overlapped with the original list of differentially expressed miRNAs, resulting in a hit rate of 28.89%. When using miRSN-DA to expand the colon cancer miRNA list, we observed an increase to 173 overlapping miRNAs. Similarly, miRSN-GOA and miRSN-PPI resulted in 170 and 173 overlapping miRNAs, respectively. However, when employing the fusion network of miRSN-DA and miRSN-GOA for miRNA list expansion, we obtained 204 overlapping miRNAs with the colon cancer miRNA set. This increased the hit rate to 64.76%, indicating a significant improvement of 35.87%. The utilization of the fused miRNA similarity network as input for the random walk with restart allowed for a more comprehensive exploration of differentially expressed miRNAs in colon cancer. This approach improved the hit rate and enhanced the power of the enrichment analysis. However, when the network fused from all three networks was used to expand the list of differentially expressed miRNAs for colon cancer, the hit rate did not improve. In fact, both the *p*-value and FDR value increased. This trend was more evident in the enrichment analysis, where the number of hit miRNAs decreased by 3, and the *p*-value and FDR increased. There are a couple of possible explanations for these observations. Firstly, miRSN-PPI contains information on only 495 miRNA-miRNA similarity pairs, whereas miRSN-DA and miRSN-GOA include a larger number of miRNAs with 1,041 and 1,063, respectively. It is likely that the miRNA similarity information in miRSN-PPI is already covered by these two networks, resulting in no improvement in the enrichment analysis after fusion with the third network. Secondly, miRSN-PPI might contain some redundant miRNA similarity information, which slightly reduces the effectiveness of the enrichment analysis when using the fusion of all three networks.

For the original differentially expressed miRNA list in colon cancer, the enrichment analysis yielded a *p*-value of $3.22e^{-23}$ and an FDR value of $1.01e^{-20}$ . In contrast, the colon cancer miRNA list expanded through MHIF-MSEA showed significantly improved results in enrichment analysis, with a *p*-value of $1.51e^{-55}$ and an FDR value of $2.43e^{-53}$ . This indicated that the enrichment analysis results for the expanded miRNA list were more pronounced. Furthermore, the experimental results presented in Tables 2, 3 demonstrated that the expansion of the original differentially expressed miRNA lists for breast cancer and hepatocellular carcinoma using the fused similarity network of miRSN-DA and miRSN-GOA yielded the highest hit counts. Simultaneously, these expansions resulted in the smallest *p*-value and FDR value. These observations suggest that the miRNA list expanded through the fused miRNA similarity network outperforms the original list in terms of enrichment analysis for breast cancer and hepatocellular carcinoma. Consequently, in the subsequent specific study and analysis of the cases, the fused network of miRSN-DA and miRSN-GOA was chosen as the miRNA-miRNA association network for the MHIF-MSEA model (see Supplementary Table S6). This fused network served as the input for the random walk with restart, which expanded the differentially expressed miRNA list. Subsequently, the MHIF-MSEA model performed enrichment analysis on the expanded differentially expressed miRNA list.

### 3.3 Case studies of miRNA set enrichment analysis

In the previous section, we have already verified that selecting the fused network of miRSN-DA and miRSN-GOA as the miRNA-miRNA association network for the MHIF-MSEA model provides optimal enrichment analysis results. To provide further validation of the effectiveness of the MHIF-MSEA model, we conducted a comprehensive and detailed analysis of the enrichment analysis results for the breast cancer and hepatocellular carcinoma cases. This analysis involved comparing the results obtained from the original differential expression list with those obtained using the expanded miRNA list generated by the MHIF-MSEA model.

**FIGURE 3:**
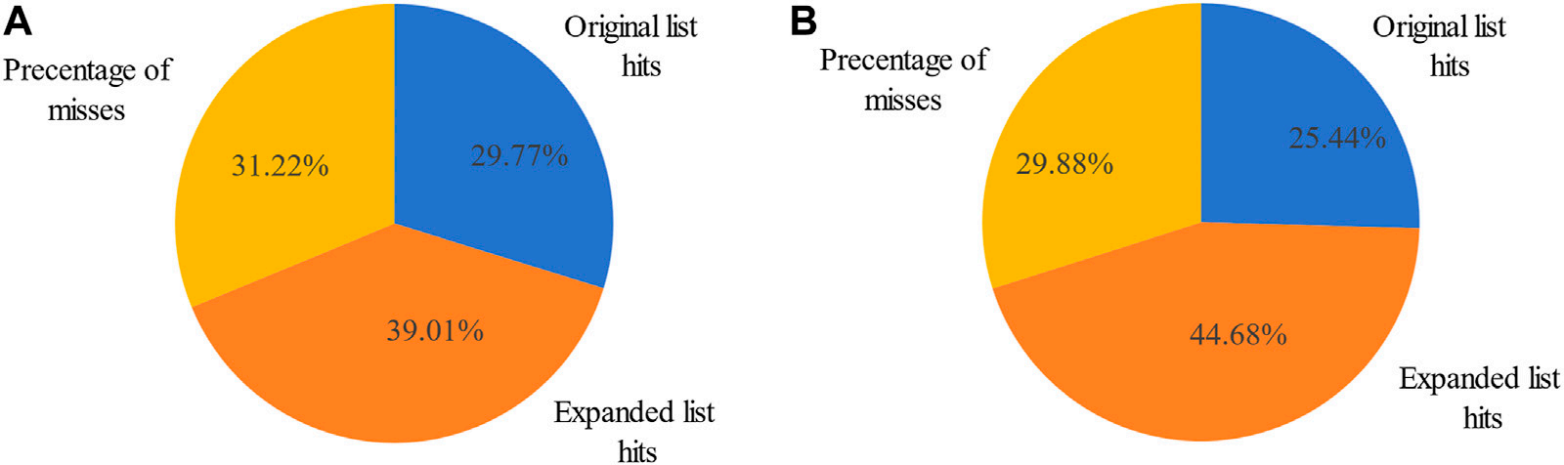
Percentage of miRNA hits in the original differentially expressed miRNA list and MHIF-MSEA expanded list for breast cancer **(A)** and hepatocellular carcinoma **(B)**.

The original breast cancer differential expression gene list overlapped with the 103 miRNAs from the breast cancer miRNA set, which consisted of 346 miRNAs, resulting in a hit rate of 29.77%. After expansion by MHIF-MSEA, the expanded miRNA list overlapped with 238 miRNAs from the breast cancer miRNA set, achieving an increased hit rate of 68.78%, representing an improvement of 39.01%, as shown in Figure 3A. In the original differential expression miRNA list, the *p*-value for breast cancer enrichment analysis was calculated to be $2.92e^{-29}$ , with an FDR value of $6.11e^{-27}$ . In contrast, the expanded miRNA list enriched by MHIF-MSEA yielded a significantly lower *p*-value of $2.17e^{-75}$ and an FDR value of $6.95e^{-73}$ . Therefore, the MHIF-MSEA expanded miRNA list showed a more pronounced enrichment for breast cancer compared to the original list.

In addition, when examining the top 15 significantly enriched disease entries in the original differentially expressed miRNA list for breast cancer, the enrichment analysis results for the list expanded by MHIF-MSEA were found to be even more significant. This is evident from the data presented in Table 4.

**TABLE 4:** Comparison of the top 15 significantly enriched diseases in breast cancer original differentially expressed miRNA list and the MHIF-MSEA expanded list.

Disease	*p*-value of origin list	FDR value of origin list	*p*-value of expanded list	FDR value of expanded list
Carcinoma, Hepatocellular	$1.72e^{-34}$	$1.08e^{-31}$	$3.47e^{-83}$	$2.22e^{-80}$
Carcinoma, Breast	$2.92e^{-29}$	$6.11e^{-27}$	$2.17e^{-75}$	$6.95e^{-73}$
Osteosarcoma	$7.18e^{-29}$	$1.13e^{-26}$	$3.22e^{-49}$	$2.29e^{-47}$
Carcinoma, Lung. Non-Small-Cell	$1.22e^{-28}$	$1.53e^{-26}$	$1.97e^{-55}$	$2.52e^{-53}$
Carcinoma, Gastric	$3.39e^{-28}$	$3.55e^{-26}$	$2.28e^{-63}$	$3.66e^{-61}$
Carcinoma, Colon	$1.23e^{-27}$	$1.10e^{-25}$	$3.16e^{-65}$	$6.75e^{-63}$
Carcinoma, Prostate	$7.96e^{-27}$	$6.24e^{-25}$	$5.60e^{-51}$	$5.12e^{-49}$
Glioma	$2.45e^{-24}$	$1.54e^{-22}$	$5.96e^{-47}$	$3.47e^{-45}$
Carcinoma, Lung	$1.96e^{-23}$	$1.12e^{-21}$	$3.52e^{-48}$	$2.26e^{-46}$
Carcinoma, Ovarian	$1.69e^{-21}$	$8.84e^{-20}$	$4.77e^{-43}$	$2.55e^{-41}$
Carcinoma, Cervical	$3.71e^{-20}$	$1.79e^{-18}$	$1.85e^{-39}$	$7.92e^{-38}$
Adenocarcinoma, Lung	$2.84e^{-19}$	$1.28e^{-17}$	$4.03e^{-36}$	$1.52e^{-34}$
Carcinoma, Pancreatic	$1.21e^{-18}$	$4.48e^{-17}$	$1.10e^{-42}$	$5.44e^{-41}$
Glioblastoma	$1.29e^{-17}$	$4.48e^{-16}$	$8.17e^{-33}$	$2.28e^{-31}$
Carcinoma, Bladder	$2.51e^{-17}$	$8.29e^{-16}$	$1.23e^{-32}$	$3.28e^{-31}$

The MHIF-MSEA model not only successfully identified all 286 significantly enriched disease entries in the original breast cancer differential expression gene list but also discovered an additional 96 disease entries enriched in the expanded list generated by MHIF-MSEA. Table 5 provides an overview of the top 10 significantly enriched disease entries uniquely identified by the MHIF-MSEA model. Notably, diseases such as Leukemia, Stroke, Idiopathic Pulmonary Fibrosis, and others were exclusively recognized by the MHIF-MSEA model.

**TABLE 5:** The top 10 significantly enriched diseases only identified by MHIF-MSEA among breast cancer differentially expressed miRNAs.

Disease	*p*-value of expanded list	FDR value of expanded list
Leukemia, Lymphocytic, Chronic, B-Cell	$2.07e^{-11}$	$1.01e^{-10}$
Stroke	$4.49e^{-8}$	$1.40e^{-7}$
Idiopathic Pulmonary Fibrosis	$2.43e^{-6}$	$5.48e^{-6}$
Focal Segmental Glomerulosclerosis	$3.15e^{-6}$	$6.99e^{-6}$
Carcinoma, Basal Cell	$5.98e^{-5}$	$1.08e^{-4}$
Pulmonary Sarcoidosis	$2.49e^{-4}$	$4.03e^{-4}$
Ischemic Diseases	$2.63e^{-4}$	$4.15e^{-4}$
Spinal Cord Injuries	$2.63e^{-4}$	$4.15e^{-4}$
Ewing’s Sarcoma	$7.58e^{-4}$	$1.13e^{-3}$
Lymphoma, Burkitt’s	$1.87e^{-3}$	$2.67e^{-3}$

Recent research studies have provided supporting evidence for the findings of the MHIF-MSEA model, demonstrating its ability to identify diseases that may be missed by traditional enrichment analysis methods with high accuracy. For instance, a study identified the HER2 pathway in breast cancer as a potential driver of pulmonary fibroblast invasion, highlighting its relevance as a target for idiopathic pulmonary fibrosis treatment and intervention (Liu et al., 2022). Moreover, a research has confirmed a significant increase in the risk of stroke in female patients with a history of breast cancer (Nilsson et al., 2005). Another study confirmed the association between chemotherapy in breast cancer patients and an elevated risk of leukemia (Cole and Strair, 2010). Additionally, a study reported a case of basal cell carcinoma following radiotherapy for breast cancer in 2021 (Marques-Antunes et al., 2021). Moreover, the associations between breast cancer and pulmonary sarcoidosis, ischemic diseases, spinal cord injuries, Ewing’s sarcoma and Lymphoma have been validated in the literature (Dong et al., 2021; Sawatzky et al., 2021; Altshuler et al., 2023). Collectively, these findings provide further confirmation of the MHIF-MSEA model’s ability to accurately identify diseases that traditional enrichment analysis methods may overlook.

The other case analysis in our study is hepatocellular carcinoma, the enrichment analysis was performed with both the original differentially expressed miRNA list and the list expanded by MHIF-MSEA. As shown in Figure 3B, the hepatocellular carcinoma disease miRNA set contained 338 miRNAs and only 86 miRNAs from the original differentially expressed miRNA list, with a hit rate of 25.44%. Conversely, the expanded miRNA list by MHIF-MSEA showed an overlap of 237 miRNAs with the hepatocellular carcinoma miRNA set, resulting in an increased hit rate of 70.12%, marking a 44.68% improvement. In the original differentially expressed miRNA list, hepatocellular carcinoma yielded a calculated *p*-value of $2.81e^{-22}$ and an FDR value of $1.74e^{-19}$ . In contrast, the hepatocellular carcinoma miRNA list expanded through MHIF-MSEA showed a calculated *p*-value of $1.50e^{-77}$ and an FDR value of $9.61e^{-75}$ . The MHIF-MSEA expanded miRNA list provided a more significant enrichment in hepatocellular carcinoma compared to the original list.

**FIGURE 4:**
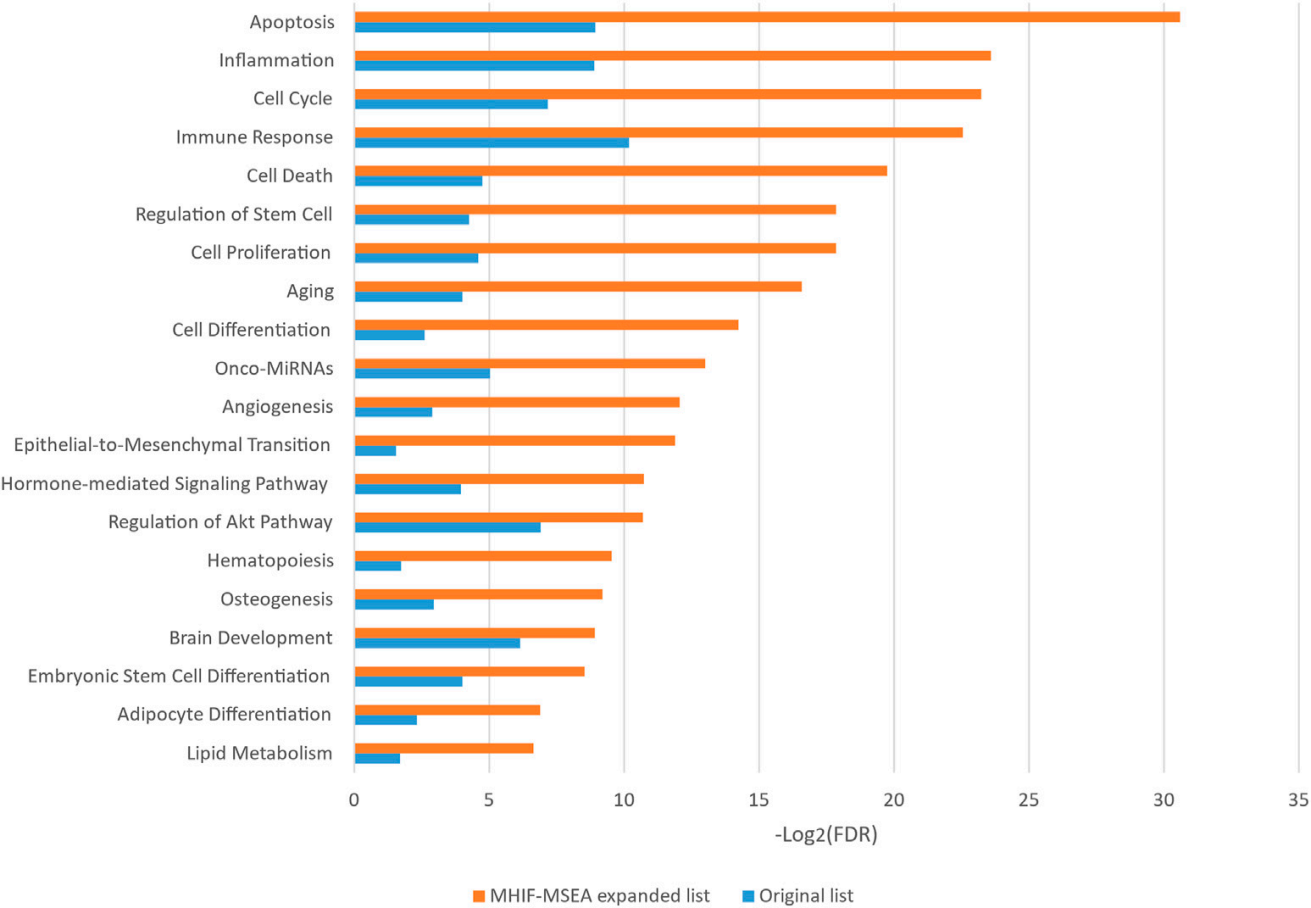
The top 20 significantly enriched functional entries exported by MHIF-MSEA among hepatocellular carcinoma differentially expressed miRNAs.

In this study, the MHIF-MSEA model was applied to investigate key biological pathways in hepatocellular carcinoma, resulting in the identification of 84 significantly enriched functional pathways. The results of the significantly enriched pathways, both for the MHIF-MSEA expanded list and the original list, are depicted in Figure 4. The findings revealed that hepatocellular carcinoma is associated with a significant enrichment of pathways related to apoptosis, inflammation, cell cycle, immune response, cell death, regulation of stem cells, cell proliferation, and more. These pathways have been validated in the existing literature and are closely linked to the occurrence and progression of hepatocellular carcinoma (Matsuda et al., 2013; Chang et al., 2018; Keenan et al., 2019; Liu et al., 2020; Demirtas and Gunduz, 2021; Hu et al., 2022). The results of the enrichment analysis not only demonstrated high consistency between the expanded list generated by MHIF-MSEA and the original list but also indicated better performance for the expanded list by exhibiting significantly higher enrichment levels.

**FIGURE 5:**
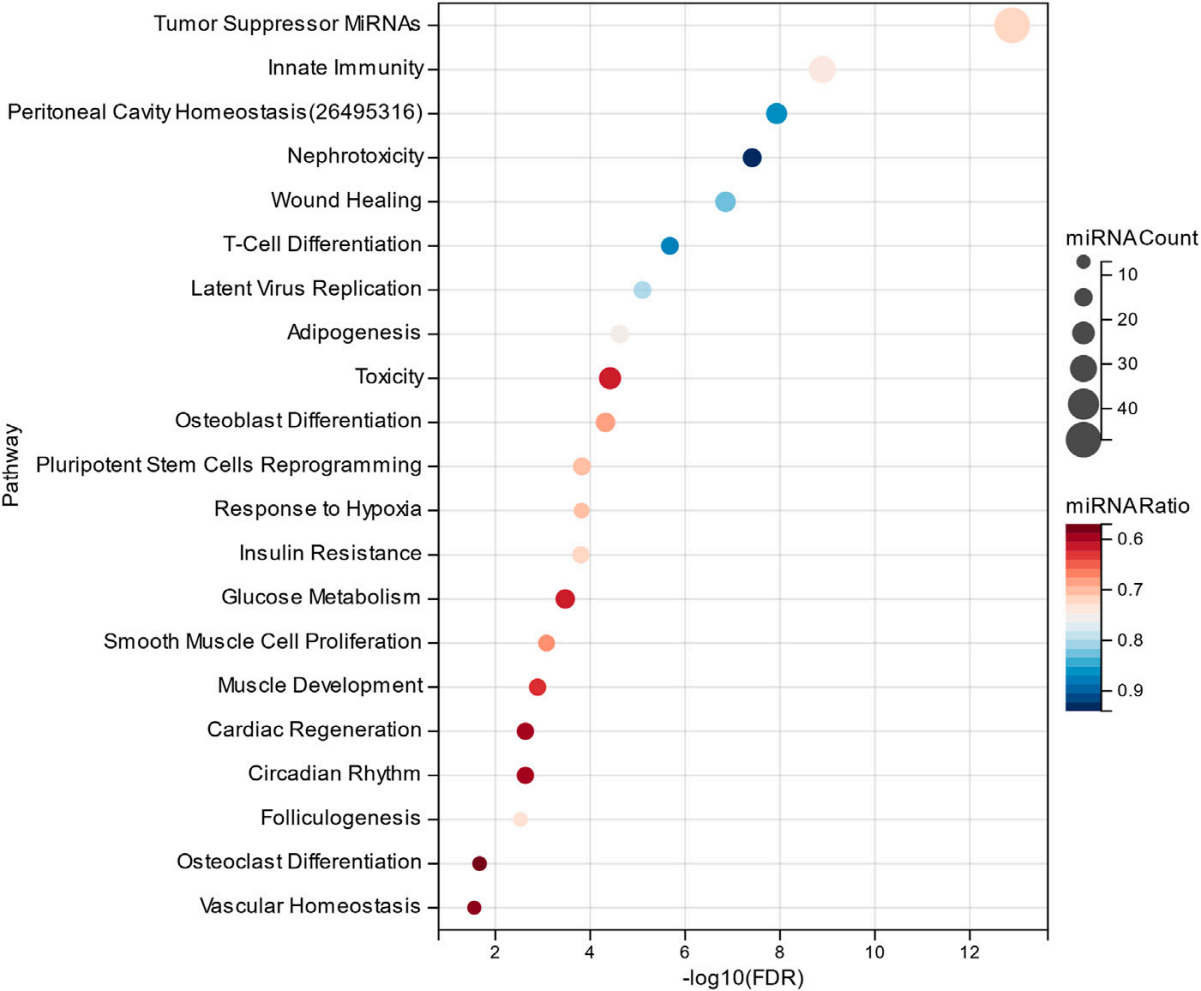
Significantly enriched functional entries only identified by MHIF-MSEA among hepatocellular carcinoma differentially expressed miRNAs.

The MHIF-MSEA model not only successfully identified all 46 significantly enriched functional pathways in the original list but also discovered an additional 21 biological pathways that were exclusively enriched in the MHIF-MSEA model. This can be observed in Figure 5, which highlights the pathways uniquely identified by MHIF-MSEA. These findings indicate that the MHIF-MSEA model has the capability to uncover novel and distinct pathways that may not be captured by traditional enrichment analysis methods. Notably, pathways such as “Tumor Suppressor MiRNAs” (FDR = $1.28e^{-13}$ ) and “Innate Immunity” (FDR = $1.29e^{-9}$ ) were exclusively identified by MHIF-MSEA.

Hepatocellular carcinoma is closely associated with alterations in glucose metabolism, as hepatocellular carcinoma cells exhibit increased glucose uptake through glucose transporters to support their high proliferation rate (Mossenta et al., 2020). Innate immune cells have been identified as key contributors to early symptoms of hepatocellular carcinoma, such as liver cirrhosis, making them a promising therapeutic target for hepatocellular carcinoma (Roderburg et al., 2020). Additionally, the significantly enriched functional pathways identified by the MHIF-MSEA model include Insulin Resistance, Adipogenesis, and Hepatotoxicity, among others. These pathways have been validated in the literature (Siddique and Kowdley, 2011; Jiang et al., 2021; Singh et al., 2023). The findings from this study demonstrate that MHIF-MSEA has the capability to identify important biological pathways that are often overlooked by conventional methods. This provides new perspectives and insights for exploring the functional roles of miRNAs and devising appropriate treatment strategies for diseases such as hepatocellular carcinoma.

## 4 Conclusion and discussion

In this study, we proposed the miRNA set enrichment analysis model based on multi-source heterogeneous information fusion (MHIF-MSEA). For this research, three distinct types of miRNA functional similarity networks were collected initially. Then, various fusion strategies were applied to combine the three miRNA functional similarity networks, selecting the best fused miRNA similarity network as the miRNA-mRNA association network. Finally, the miRNA-miRNA association network was analyzed using a random walk with restart algorithm to identify miRNAs closely related to the miRNA list of interest. This expanded the original miRNA list for subsequent enrichment analysis. The MHIF-MSEA model offers a novel approach to miRNA functional enrichment analysis, overcoming the limitations of existing methods. The miRNA-mRNA association network was constructed using the fusion network of miRSN-DA and miRSN-GOA, which was identified as the optimal choice for the MHIF-MSEA model based on three cancer cases (colon cancer, breast cancer, and hepatocellular carcinoma). Further analysis of the enrichment results from breast cancer and hepatocellular carcinoma confirmed the effectiveness and reliability of the MHIF-MSEA model.

Although MHIF-MSEA has demonstrated satisfactory results in functional enrichment analysis, there are opportunities for further improvement. Firstly, the construction of the miRNA-miRNA association network in this study only considered three miRNA functional similarity networks, neglecting other types of miRNA functional annotation networks, such as expression-based miRNA functional annotation networks. This omission may lead to the loss of relevant miRNA association information. To address this limitation, it is essential to collect and integrate diverse heterogeneous miRNA functional annotation networks. By incorporating a broader range of miRNA functional information sources, a more comprehensive understanding of miRNA associations can be achieved, thereby better representing the intricate network relationships between miRNAs. Furthermore, the method used to integrate miRNA similarity networks in this study was relatively simplistic and requires further refinement. To enhance the accuracy and comprehensiveness of miRNA functional enrichment analysis, advanced techniques like Graph Convolutional Networks (GCN) can be employed to fuse different types of miRNA functional annotation networks. GCN-based approaches can effectively capture the complex association information between miRNAs, leading to more comprehensive and accurate results in miRNA functional enrichment analysis.

In summary, there is still potential for improvement in MHIF-MSEA regarding functional enrichment analysis. Future versions of MHIF-MSEA should consider incorporating additional categories of miRNAs, integrating various miRNA functional information sources into the miRNA-miRNA network, and developing more efficient methods for expanding miRNA lists of interest. These advancements will contribute to enhancing the performance and capabilities of MHIF-MSEA in miRNA functional enrichment analysis.

## Data Availability

The original contributions presented in the study are included in the article/Supplementary Material, further inquiries can be directed to the corresponding authors.
